# Predicting Alzheimer’s progression in MCI: a DTI-based white matter network model

**DOI:** 10.1186/s12880-024-01284-7

**Published:** 2024-05-03

**Authors:** Qiaowei Song, Jiaxuan Peng, Zhenyu Shu, Yuyun Xu, Yuan Shao, Wen Yu, Liang Yu

**Affiliations:** 1 Center for Rehabilitation Medicine, Department of Radiology, Affiliated People’s Hospital, Zhejiang Provincial People’s Hospital, Hangzhou Medical College, Hangzhou, Zhejiang China; 2 Jinzhou medical university, Jinzhou, China

**Keywords:** Radiomics, Diffusion Tensor Imaging, Mild cognitive impairment, Alzheimer’s disease, White matter

## Abstract

**Objective:**

This study aimed to identify features of white matter network attributes based on diffusion tensor imaging (DTI) that might lead to progression from mild cognitive impairment (MCI) and construct a comprehensive model based on these features for predicting the population at high risk of progression to Alzheimer’s disease (AD) in MCI patients.

**Methods:**

This study enrolled 121 MCI patients from the Alzheimer’s Disease Neuroimaging Initiative (ADNI). Among them, 36 progressed to AD after four years of follow-up. A brain network was constructed for each patient based on white matter fiber tracts, and network attribute features were extracted. White matter network features were downscaled, and white matter markers were constructed using an integrated downscaling approach, followed by forming an integrated model with clinical features and performance evaluation.

**Results:**

APOE4 and ADAS scores were used as independent predictors and combined with white matter network markers to construct a comprehensive model. The diagnostic efficacy of the comprehensive model was 0.924 and 0.919, sensitivity was 0.864 and 0.900, and specificity was 0.871 and 0.815 in the training and test groups, respectively. The Delong test showed significant differences (*P* < 0.05) in the diagnostic efficacy of the combined model and APOE4 and ADAS scores, while there was no significant difference (*P* > 0.05) between the combined model and white matter network biomarkers.

**Conclusions:**

A comprehensive model constructed based on white matter network markers can identify MCI patients at high risk of progression to AD and provide an adjunct biomarker helpful in early AD detection.

**Supplementary Information:**

The online version contains supplementary material available at 10.1186/s12880-024-01284-7.

## Introduction

Alzheimer’s disease (AD) is the most common form of dementia, accounting for 50–75% of all patients with AD [1]. Cognitive impairment is the preliminary stage of AD, and mild cognitive impairment (MCI) is an intermediate state between normal aging and AD. Some patients can progress to AD if adequate treatment is not provided at the MCI stage. Therefore, early diagnosis and intervention are crucial for MCI patients at high risk of progression to AD.

The advancement of neuroimaging techniques has facilitated the non-invasive identification of alterations in specific brain regions that may underlie early mild cognitive impairment (MCI) [2]. Presently, magnetic resonance imaging (MRI) has emerged as the primary neuroimaging modality for assessing structural changes in the brain associated with distinct clinical manifestations observed in individuals with MCI and AD [3]. Since the cortex is generally considered the principal repository of cognitive function, most previous MRI structural image studies on cognition have focused on the gray matter of the cortex [4]. However, available evidence suggests that white matter, an essential component of the subcortex, is relevant to cognitive functions [5]. White matter mainly comprises glial cells and myelin sheaths, and its primary function is to transmit neural impulses and messages. In addition, white matter connects different brain regions through a series of fiber bundles. Advanced diffusion magnetic resonance imaging (dMRI), which is currently the only in vivo non-invasive assessment of white matter fiber tracts, has become an important means of studying white matter structure and obtaining imaging biomarkers, whereas diffusion tensor imaging (DTI) features such as fractional anisotropy (FA) and mean diffusivity (MD) can quantify the white matter microstructure and are widely used in MCI or AD studies [6–8]. However, “cross-fibers” in white matter lead to uncertainty in the biological interpretation of these findings [9].

Recent advances in MRI techniques have enabled the investigation of network connectivity in patients with MCI. Functional magnetic resonance imaging (fMRI) has become a common tool to in vestigate functional connectivity, which is a statistical measure of correlation between neuronal activities [10]. A growing number of studies have reported reduced functional connectivity in the default mode network (DMN), fronto-parietal net work, and thalamo-cortical network in patients with MCI [11–13]. At present, white matter (WM) network connection has drawn increasing attention among studies of MCI. The “disconnection hypothesis” has been proposed, which postulates that WM microstructure lesions result in the inter ruption of communication between cortical regions, thereby resulting in poorer cognitive performance [14]. On the other hand, structural networks based on DTI have been widely employed in the field of neuroscience as a potential approach for investigating MCI. Previous studies have shown that the connectivity characteristics of brain networks based on white matter fiber bundles are closely related to cognitive function [15, 16], and DTI-based brain network connectivity has been demonstrated to possess predictive capabilities for prodromal AD patients [17]. Additionally, Savarraj et al reported that identified the ‘right anterior cingulum’ and ‘right frontal superior medial’ as early predictors of AD within the biological network constructed using white matter tracts [18]. Based on the aforementioned research findings, we hypothesis that the topological metrics of WM structural networks in patients with MCI has the potential to identify the population at high risk of progression to AD.

The primary purpose of this study is to analyze the network attribute features related to the progression of MCI disease from the white matter network and then combine these features with relevant clinical features to build an integrated model to predict high-risk MCI patients for progression to AD, which can become a crucial tool for early diagnosis and intervention of MCI.

## Materials and methods

### Demographic information

The case data included in this study were all from the public dataset available on the Alzheimer’s Disease Neuroimaging Initiative (ADNI) official website (https://adni.loni.usc.edu/data-samples/access-data/#access_data), and ethical review information regarding ADNI data is available on the website. A total of 121 patients diagnosed with MCI at the baseline stage were included, 32 of whom progressed to AD during the 4-years follow-up period and were placed in the progression group. The inclusion criteria were as follows: all patients with an initial diagnosis of MCI were followed up for 4 years, underwent MRI examinations, and had complete clinical data. The exclusion criteria were as follows: (1) the original MRI DICOM file was incorrect and we were unable to extract network features; and (2) biological indicator and scale evaluation data were missing. The cases were randomly divided into a training set (*n* = 84) and a testing set (*n* = 37) in a 7:3 ratio. The training set was used to build the model, and the testing set was used to validate the performance of the model. In addition, this study also collected relevant demographic data, including age, gender, and education level, and clinical data, including neural scale information such as MMSE (Mini-Mental State Examination), CDR (Clinical Dementia Rating), ADAS (Alzheimer’s Disease Assessment Scale), and APOE4, as complementary features for the model construction.

### Data pre-processing and network attribute feature acquisition

All patients underwent DTI examination, which was performed using a 3.0T MRI scanner (GE Company). The DTI images were first pre-processed and analyzed using FSL software, with steps including cranial stripping, eddy current correction, head motion correction, and adjustment of diffusion gradient orientation. Before fiber tracking, poor-quality subjects were eliminated by a quality control program. Subsequently, the corrected images and reoriented b-value tables were imported into DSI Studio (http://dsi-studio.labsolver.org). The focused ion beam (FIB) reconstruction algorithm of q-space diffeomorphic reconstruction (QSDR) was used to keep the tracked fibers in standard space. The deterministic fiber tracking algorithm and an enhanced tracking strategy were used to improve reproducibility. The parameter settings for fiber tracking included diffusion sampling length ratio of 1.25; fiber bundle angle threshold of 45 degrees; step size randomly chosen from 0.5 voxels to 1.5 voxels; filtering fiber bundles with lengths less than 30 mm or more than 300 mm with seed points of 1,000,000 and tracking fiber travel in this way. Each patient got the respective DTI brain network, with each network corresponding to a 120 × 120 matrix. Finally, the AAL2 atlas was used as a template [19], with specific brain regions as nodes and inter-node connectivity index FA as edges, to construct the corresponding brain network and calculate the network attribute values. Finally, 960 feature values were extracted to reflect the topological attribute changes of the DTI brain network. Detailed of feature and DTI scan information can be found in supplementary materials.

### Feature dimensionality reduction and white matter network biomarkers construction

To exclude irreducible, redundant, and irrelevant features of the initial set from the extracted network attribute features, we used Max-Relevance and Min-Redundancy (mRMR) [20], Least absolute shrinkage and selection operator (LASSO) [21], and Gradient Boosting Decision Tree (GBDT) set dimensionality reduction methods for the extracted feature sets in the training set [22]. Then, the remaining features were used to construct white matter network biomarkers. Support vector machine (SVM) is the most commonly used machine learning algorithm and has been proven to predict the future diagnosis of AD. Therefore, we used the SVM algorithm to construct the white matter network biomarker [23]. The quantitative values calculated for each case based on this biomarker reflected the probability of MCI progressing to AD. The area under the curve (AUC) of the receiver operating characteristics (ROC) curve was used to evaluate the accuracy of the white matter network biomarkers in the training and test sets. The detailed steps of feature reduction and machine learning are described in the supplementary material.

### Construction and validation of the combined model

**Fig. 1:**
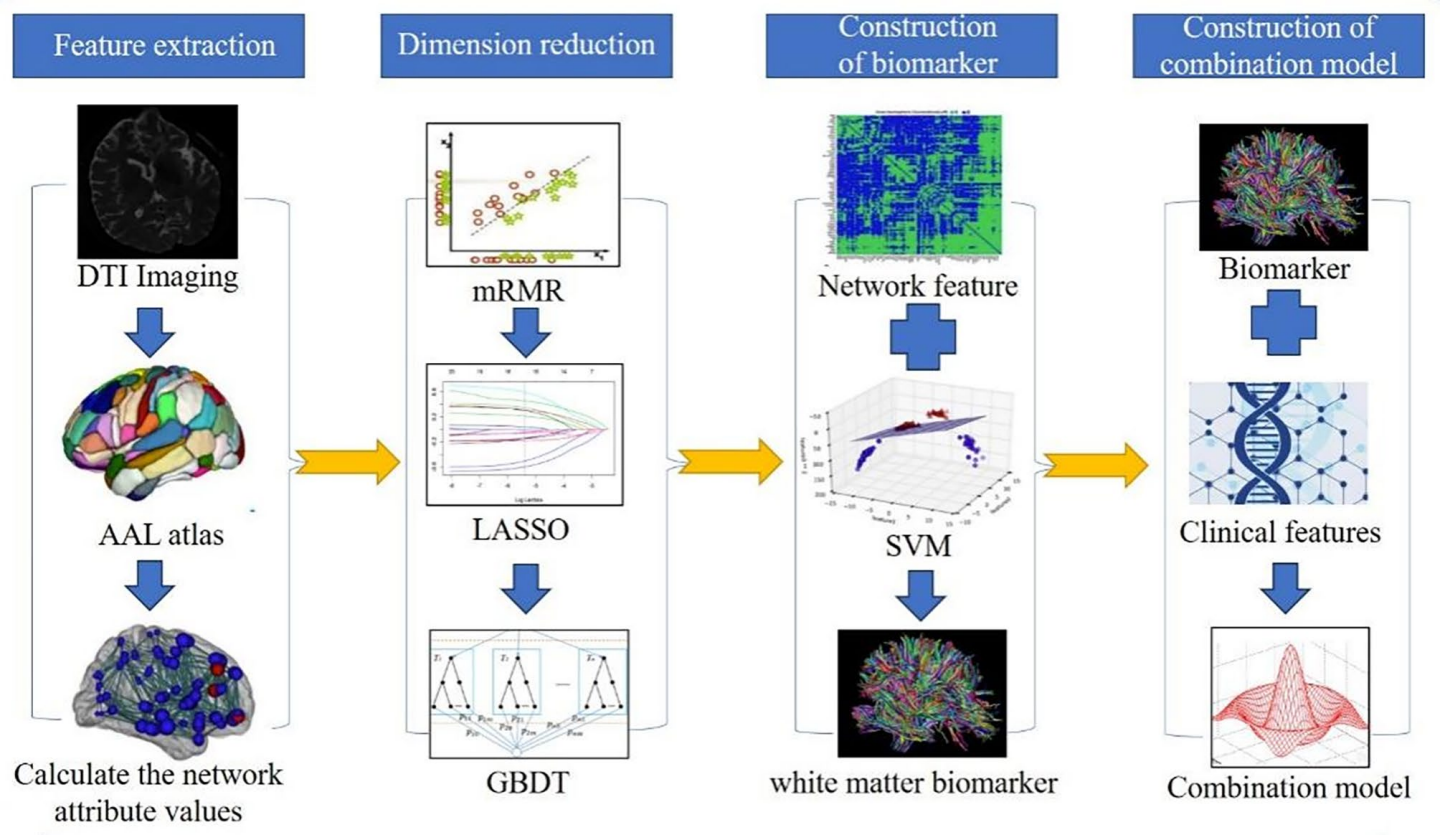
Combined model construction process diagram

Since nonlinear models perform more robustly and can better utilize the information in the non-imaging data [24], the present study used logistic regression to construct the combined model. A backward stepwise selection method based on the Akaike information criterion (AIC) stopping rule was used to select independent predictors from clinical features and white matter network biomarkers in the training group, and the combined model was built, as shown in Fig. 1. To validate the improvement in model performance after including white matter network markers, we used the area under the receiver operating characteristic (ROC) curve (AUC) to evaluate the performance of different independent predictors, and the DeLong test was used to determine the difference between the combined model and other independent predictors. In addition, we used the Hosmer-Lemeshow test to analyze the fit of the combined model superiority and visualized it using calibration curves. Finally, the risk of progression to AD was calculated for each patient based on the model, after which the cases in the training and test groups were divided into low- and high-risk groups based on the cut-off values of the ROC curves, and the difference in the progression rate of MCI was examined using Kaplan-Meier survival curve analysis for both groups.

### Statistical analysis

Statistical analysis was conducted using R statistical software (version 3.6.3; http://www.Rproject.org), SPSS (version 24.0), and MedCalc (version 11.2). The *t*-test, Mann-Whitney U test, *X*^*2*^ test, or Fisher’s exact test were used to compare continuous and categorical variables. *P* < 0.05 was considered statistically significant.

## Results

### Comparison of clinical characteristics

There was no statistically significant difference in all clinical data information between the training and test groups (*P* > 0.05). There were significant differences in CDR and ADAS scores between the MCI stable group and the progression group in both the training and test groups (*P* < 0.05). In addition, there was a statistically significant difference in MMSE scores between the stable and progression groups in the training group (*P* < 0.05), while there was no statistical difference in other clinical data (*P* > 0.05). See Table 1 for details.

**Table 1:** Comparative analysis of clinical data in the training and test sets

Characteristics		ALL cohort (*n* = 121)	Training cohort (*n* = 84)			Test cohort (*n* = 37)			Training VS Test *P* value
			MCI stable (*n* = 62)	MCI progression (*n* = 22)	*P*value	MCI stable (*n* = 27)	MCI progression (*n* = 10)	*P*value	
Gender (n, %)	Male	73 (60.33)	39 (62.9)	11 (50)	0.289	16 (59.26)	7 (70.00)	0.71	0.785
	Female	48 (39.67)	23 (37.1)	11 (50)		11 (40.74)	3 (30.00)		
APOE4 (n, %)	negative	56 (46.28)	29 (46.77)	8 (36.36)	0.398	19 (70.37)	0 (00.00)	0.001	0.458
	positive	65 (53.72)	33 (53.23)	14 (63.64)		8 (29.63)	10 (100.00)		
Age (year)		72.74 ± 7.3	71.1 ± 7.86	73.68 ± 7.38	0.072	73.95 ± 5.89	73.17 ± 4.52	0.707	0.254
MMSE		27.88 ± 1.65	28.06 ± 1.76	27 ± 1.51	0.014*	28.41 ± 1.12	27.20 ± 1.69	0.057	0.323
CDRS		1.37 ± 0.78	1.27 ± 0.81	1.73 ± 0.69	0.019*	1.09 ± 0.50	1.95 ± 0.90	0.015*	0.685
ADAS		9.65 ± 4.61	8.66 ± 3.43	13.49 ± 5.84	0.001*	7.81 ± 3.83	12.30 ± 4.62	0.005*	0.328
Education (years)		15.88 ± 2.68	15.73 ± 2.6	16.45 ± 2.76	0.269	16.04 ± 2.81	15.10 ± 2.77	0.372	0.803

Note. ADAS, Alzheimer’s disease assessment scale; APOE4, apolipoprotein E 4; CDRS, clinical dementia rating scale; MMSE, mini-mental state examination. * indicates *p* < 0.05

### Dimension reduction and biomarker construction of white matter network attribute features

**Fig. 2:**
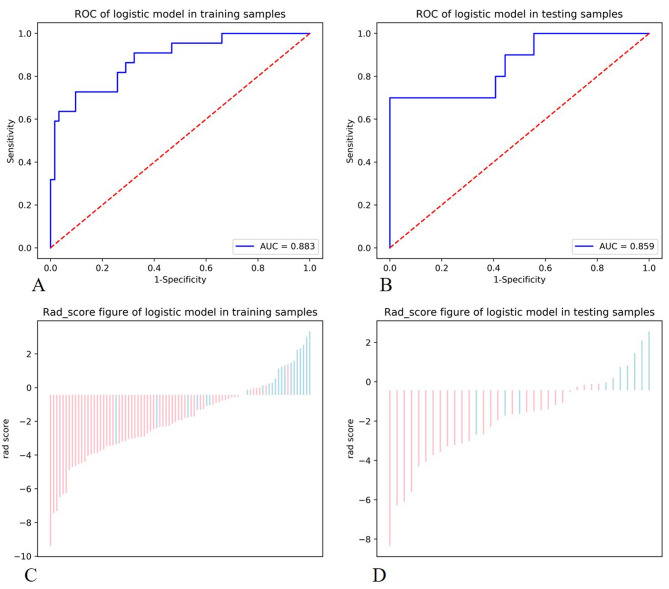
Figures **A** and **B** show the diagnostic effectiveness of white matter network biomarkers in the training and test groups, respectively. Figures **C** and **D** show the classification effectiveness of markers in training and test groups, with values less than 0 indicating stable cases of MCI, values greater than 0 indicating progressive cases of MCI, blue indicating progressive cases, and red indicating stable cases

After dimensionality reduction, five network attribute features were finally selected for the construction of white matter biomarkers, including pagerank_ centrality_ Fusiform_ R, betweenness_ centrality_ Occipital_ Inf_ L, betweenness_ centrality_ Temporal_ Inf_ L, betweenness_ centrality_ Postcentral_ L, and pagerank_ centrality_ Occipital_ Mid_ L. The white biomarkers constructed based on the above five features had good predictive performance in training and test sets, with AUC of 0.883 and 0.859, specificity of 0.903 and 0.852, and sensitivity of 0.782 and 0.7, respectively, as shown in Fig. 2.

### Construction and validation of the combined model

**Fig. 3:**
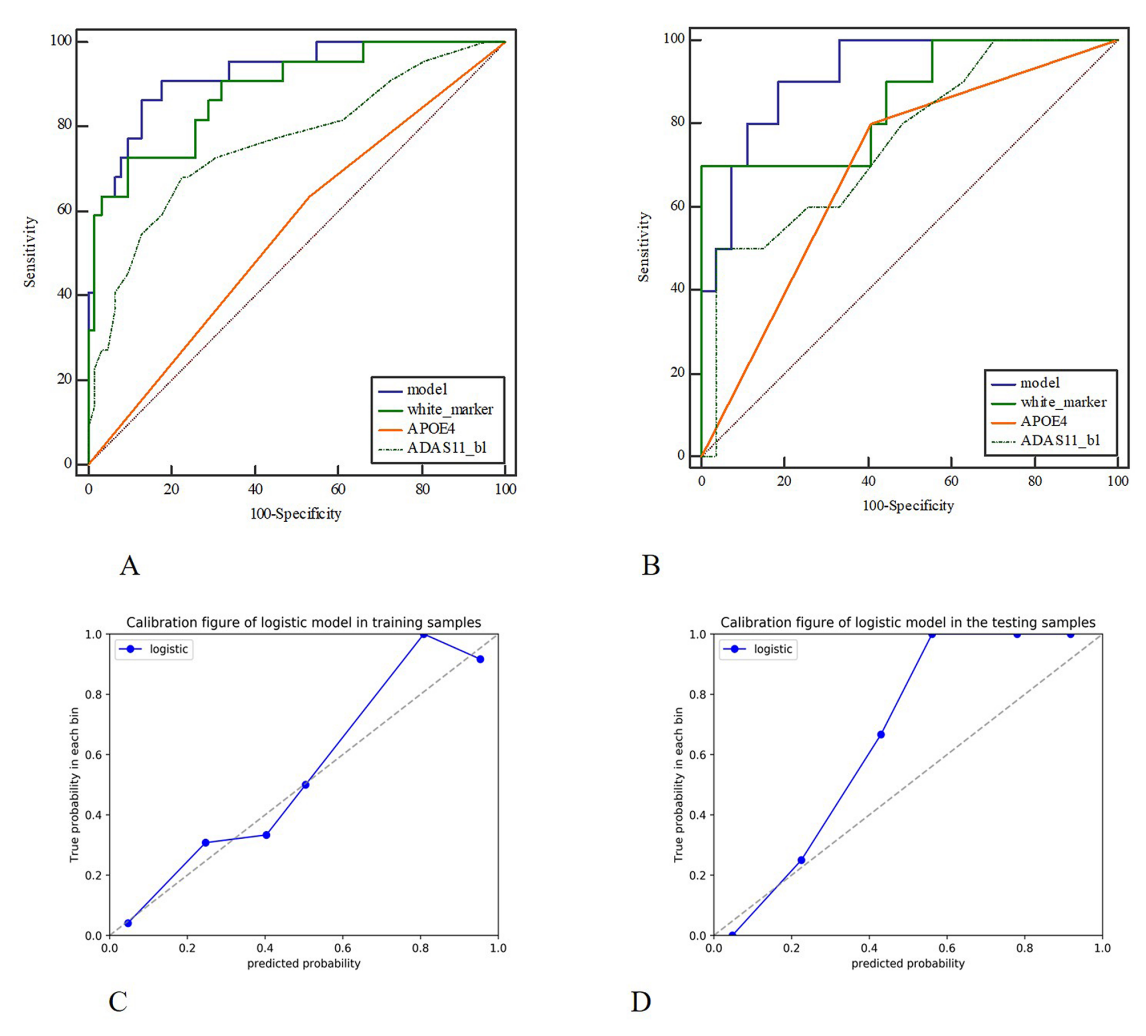
Figures **A** and **B** show the diagnostic effectiveness of the combined model and other predictive factors in the training and test groups, while Figures C and D show the calibration curves of the model in the training and test groups

**Fig. 4:**
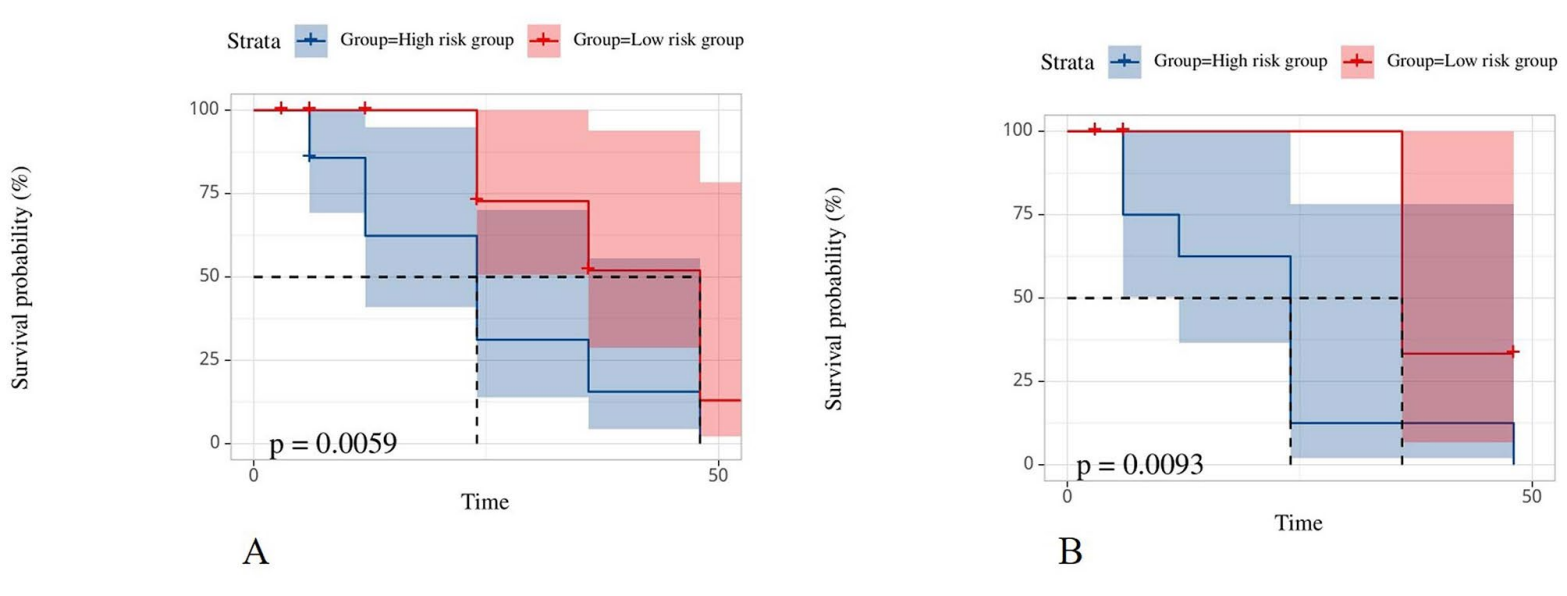
The survival curve analysis of the low- and high-risk groups based on the combined model classification in the training and test groups, respectively

The stepwise logistic regression analysis showed that APOE4 and ADAS scores and white matter network biomarkers were independent predictors of MCI progression, and a combined model was constructed, as shown in Table 2. The Hosmer – Lemeshow test showed that the combined model did not overfit (*P* > 0.05), and the calibration curve showed that the prediction efficiency of the combined model was consistent with the actual MCI progression state. The ROC curves showed that the AUC of the combined model was 0.924 and 0.919, sensitivity was 0.864 and 0.900, and specificity was 0.871 and 0.815 in the training and test groups, respectively. The Delong test showed significant differences (*P* < 0.05) in the diagnostic efficacy of the combined model and APOE4 and ADAS scores in the training and the test groups, while there was no significant difference (*P* > 0.05) between the combined model and white matter network biomarkers, as shown in Fig. 3; Table 3. Patients were divided into low-risk and high-risk groups according to the best cut-off value of 1.004. Kaplan-Meier survival curve analysis showed a statistically significant difference in MCI progression time between the low- and high-risk groups in both the training and test groups (Fig. 4).

**Table 2:** Results of univariate and multivariate logistic regression analyses

Variable	Univariate logistic regression		Multivariate logistic regression	
	OR (95%CI)	*P* value	OR (95%CI)	*P* value
Gender	1.258 (0.555, 2854)	0.583	NA	NA
APOE4	3.512 (1.425, 8.658)	0.006	4.421 (1.240,15.765)	0.022
Age (year)	1.060 (0.999, 1.125)	0.054	NA	NA
MMSE	0.666 (0.516, 0.859)	0.002	NA	NA
CDRS	2.619 (1.513, 4.532)	0.001	NA	NA
ADAS	1.276 (1.142, 1.426)	0.000	1.157 (1.017,1.317)	0.026
Education (years)	1.030 (0.885, 1.199)	0.701	NA	NA
White Marker	2.443 (1.715, 3.480)	0.000	303.471 (28.942, 3182.065)	0.000

Note. ADAS, Alzheimer’s disease assessment scale; APOE4, apolipoprotein E 4; CDR, clinical dementia rating scale; MMSE, mini-mental state examination

**Table 3:** Performance of independent predictors and model in training and testing cohorts

Characteristics	Training cohort				Test cohort			
	AUC	Sensitivity	Specificity	*P*value	AUC	Sensitivity	Specificity	*P* value
Multimodal combinatorial model	0.924	0.864	0.871	NA	0.919	0.900	0.815	NA
White Marker	0.883	0.782	0.903	0.258^a^	0.859	0.7	0.852	0.306^a^
ADAS	0.76	0.682	0.774	0.002^b*^	0.757	0.500	0.963	0.046^b*^
APOE4	0.552	0.636	0.468	*p*<0.001^c*^	0.696	0.800	0.593	0.024^c*^

Note: a, b, and c indicate the comparison of diagnostic performance between the multimodal combination model and white marker, ADAS, and APOE4, respectively. * indicates *p* < 0.05

## Discussion

We performed a systematic and quantitative review of predicting the evolution of the clinical status of MCI patients over four years. Our results showed that the prediction performance of white matter network biomarkers was significantly better than the scale and the APOE4 gene alone, indicating that people with MCI progression were more sensitive to changes in the white matter structure network properties in the early stage than other clinical features. Furthermore, the combined model with these feature types significantly improved the predictive performance, providing a valuable tool for clinically screening potentially AD high-risk populations.

In this study, we identified five network attribute features, of which the Pagerank centrality was a node centrality indicator, which defines the importance of a node as the degree to which a node is connected to other vital nodes [25], while the Fusiform_ R node and Occidental_ Mid_L node showed essential alterations in the right fusiform gyrus and the left occipital brain region during the progression of MCI. In addition, betweenness_ Centrality was a measure of node centrality based on the role of nodes as “bridges” in a network [26], indicating the importance or influence of this node in connecting other nodes in the network. These nodes may reflect changes in brain network function in early mild cognitive impairment, which is also consistent with the results of Zhang et al.; namely, there is a correlation between white matter network disruption and mild cognitive impairment [27]. These results confirm that the structural network in MCI patients is associated with deterioration in disease progression and demonstrate that predicting the future values of the biomarkers or the images is significant for clinical decision support systems to be adopted in practice.

In this study, we also confirmed that APOE4 and ADAS scores participated in constructing the model as independent predictors. These results are similar to those reported by Li et al., who, through a meta-analysis of 60 cohort studies, confirmed that the above two were the risk factors for MCI progression to AD [28]. Our findings also show that using the APOE4 gene, ADAS score, or white matter biomarkers can predict whether MCI will progress to dementia. However, these indicators also have certain limitations. Although the APOE4 gene is associated with the risk of dementia, not all individuals carrying the APOE4 gene develop dementia [29], which may also be a possible reason for its poor specificity as a predictor. The ADAS score can reflect the level of cognitive function. However, as a subjective quantitative measure, it cannot directly reflect changes in neuropathology [30]. Furthermore, although the diagnostic performance of white matter biomarkers is close to that of the combined model and can also reflect some neuropathological changes, their changes may represent a relatively lengthy process of white matter microstructure transformation, which cannot immediately reflect whether patients with MCI will progress to dementia. The combined model constructed by integrating the above three shows the highest diagnostic performance and achieves high sensitivity and specificity. Therefore, the combined model using these indicators can more comprehensively and accurately reflect whether MCI patients will progress to dementia, which may also provide new ideas and methods for dementia prevention and treatment. The model’s effectiveness in this study was comparatively high compared to previous similar studies. Lin et al. used gray matter density changes and atrophy patterns in longitudinal magnetic resonance structural images to predict MCI conversion, with an AUC of 0.984 [31]. They had a higher accuracy than our study; however, they only selected a specific slice of the brain for analysis. Our study is based on the structural network of the whole brain, which is more comprehensive. In another similar study, Sidra Minhas et al. constructed a prediction model using MRI-derived biomarkers, including brain volume, surface area, and cortical thickness of brain regions obtained after cortical segmentation, combined with some neuropsychological scales. The diagnostic ability of their model was 0.889 and 0.881 during a one-year and two-year follow-up period, respectively [32], while the diagnostic efficiency of our model was 0.919, which further suggests that structural network features are more suitable for slow-progressing disease state transitions such as AD than structural and morphological features. In particular, the follow-up period of this study was four years. Therefore, the results of this study also provide new insights for better prediction of MCI transitions.

The study also has certain limitations. Firstly, this is a single-center retrospective study. Prospective and multicenter studies are needed to verify the feasibility and effectiveness of the constructed model in a more extensive and diverse sample. Secondly, there is some uncertainty in the feature selection and modeling methods, and white matter network attribute features have high dimensions and complexity. Therefore, selecting and combining these features and selecting appropriate modeling methods still require more research. Finally, this study did not analyze the potential correlation between white matter network attribute features and cognitive function, which may limit the clinical applications of the constructed model. In the future, we will consider how these features reflect different types of cognitive impairment and their progression.

This study provides strong support for the prediction and diagnosis of the progression of MCI diseases using white matter network attribute features, which helps to understand the mechanism of disease progression. Moreover, a combined model based on the white matter networks attribute features provides a valuable tool for the clinical screening of high-risk MCI patients and a reference basis for developing more accurate diagnosis and treatment plans for cognitive impairment-related diseases.

### Electronic supplementary material

Below is the link to the electronic supplementary material.

Supplementary Material 1

## Data Availability

The datasets generated during and/or analysed during the current study are available from the corresponding author on reasonable request. The case data included in this study were all from the public dataset available on the Alzheimer’s Disease Neuroimaging Initiative (ADNI) official website (https://adni.loni.usc.edu/data-samples/access-data/#access_data).

## Abbreviations

DTI: Diffusion tensor imaging
MCI: Mild cognitive impairment
AD: Alzheimer’s disease
ADNI: Alzheimer’s Disease Neuroimaging Initiative
FA: Fractional anisotropy
MD: Mean diffusivity
ABA: Atlas-based analysis
mRMR: Max-Relevance and Min-Redundancy
GBDT: Gradient Boosting Decision Tree
MMSE: Mini-Mental State Examination
CDR: Clinical Dementia Rating
ADAS: Alzheimer’s Disease Assessment Scale
SVM: Support vector machine
AIC: Akaike information criterion
LASSO: Least absolute shrinkage and selection operator
