# Supplementary material for: Predicting Alzheimer’s progression in MCI: a DTI-based white matter network model
Source: BMC Med Imaging. 2024 May 3;24:103. doi: 10.1186/s12880-024-01284-7 (PMC11067201; doi:10.1186/s12880-024-01284-7)
Supplement: Supplementary file 1 — Supplementary Material 1 [file 12880_2024_1284_MOESM1_ESM.docx]

**Supplementary material**

1. **DTI Brain Network Attribute Features**

Each feature of the DTI brain network represents the network attributes of each brain region. The brain regions are strictly divided according to the standard brain atlas AAL2, and the network attributes represent graph theory topology attributes. The specific related names and meaning are shown in Tables S1 and S2.

1. **Parameters of MRI images.**

All patients underwent DTI examination, which was performed using a 3.0T MRI scanner (GE Company). DTI collection uses GRE-EPI sequence, with the following parameters: FOV=240 x 240mm, TR=14884 ms, TE=101.5 ms; Layer thickness=4 mm, matrix=120 x 120, diffusion gradient direction 30, b-value=1000 s/mm2, voxel size=2mm x 2mm x 2mm, a total of 46 layers were collected.

**Table S1. The information of Automated Anatomical Labeling**

| Precentral_L | Hippocampus_L | Thalamus_L |
| --- | --- | --- |
| Precentral_R | Hippocampus_R | Thalamus_R |
| Frontal_Sup_2_L | ParaHippocampal_L | Heschl_L |
| Frontal_Sup_2_R | ParaHippocampal_R | Heschl_R |
| Frontal_Mid_2_L | Amygdala_L | Temporal_Sup_L |
| Frontal_Mid_2_R | Amygdala_R | Temporal_Sup_R |
| Frontal_Inf_Oper_L | Calcarine_L | Temporal_Pole_Sup_L |
| Frontal_Inf_Oper_R | Calcarine_R | Temporal_Pole_Sup_R |
| Frontal_Inf_Tri_L | Cuneus_L | Temporal_Mid_L |
| Frontal_Inf_Tri_R | Cuneus_R | Temporal_Mid_R |
| Frontal_Inf_Orb_2_L | Lingual_L | Temporal_Pole_Mid_L |
| Frontal_Inf_Orb_2_R | Lingual_R | Temporal_Pole_Mid_R |
| Rolandic_Oper_L | Occipital_Sup_L | Temporal_Inf_L |
| Rolandic_Oper_R | Occipital_Sup_R | Temporal_Inf_R |
| Supp_Motor_Area_L | Occipital_Mid_L | Cerebelum_Crus1_L |
| Supp_Motor_Area_R | Occipital_Mid_R | Cerebelum_Crus1_R |
| Olfactory_L | Occipital_Inf_L | Cerebelum_Crus2_L |
| Olfactory_R | Occipital_Inf_R | Cerebelum_Crus2_R |
| Frontal_Sup_Medial_L | Fusiform_L | Cerebelum_3_L |
| Frontal_Sup_Medial_R | Fusiform_R | Cerebelum_3_R |
| Frontal_Med_Orb_L | Postcentral_L | Cerebelum_4_5_L |
| Frontal_Med_Orb_R | Postcentral_R | Cerebelum_4_5_R |
| Rectus_L | Parietal_Sup_L | Cerebelum_6_L |
| Rectus_R | Parietal_Sup_R | Cerebelum_6_R |
| OFCmed_L | Parietal_Inf_L | Cerebelum_7b_L |
| OFCmed_R | Parietal_Inf_R | Cerebelum_7b_R |
| OFCant_L | SupraMarginal_L | Cerebelum_8_L |
| OFCant_R | SupraMarginal_R | Cerebelum_8_R |
| OFCpost_L | Angular_L | Cerebelum_9_L |
| OFCpost_R | Angular_R | Cerebelum_9_R |
| OFClat_L | Precuneus_L | Cerebelum_10_L |
| OFClat_R | Precuneus_R | Cerebelum_10_R |
| Insula_L | Paracentral_Lobule_L | Vermis_1_2 |
| Insula_R | Paracentral_Lobule_R | Vermis_3 |
| Cingulate_Ant_L | Caudate_L | Vermis_4_5 |
| Cingulate_Ant_R | Caudate_R | Vermis_6 |
| Cingulate_Mid_L | Putamen_L | Vermis_7 |
| Cingulate_Mid_R | Putamen_R | Vermis_8 |
| Cingulate_Post_L | Pallidum_L | Vermis_9 |
| Cingulate_Post_R | Pallidum_R | Vermis_10 |

**Table S2. The Meaning of Network Node Attribute in Graph Theory**

| degree | Degree refers to the number of connections between a point and other points in the network. Degree Distribution refers to the probability distribution of the number of degrees of each point in the whole network. |
| --- | --- |
| strength | Node strength accounting calculates the number of neighbors of a given node, taking into account the connection weight; The strength of the node is defined as the sum of the weights of all edges connected to it |
| cluster coefficient | Clustering coefficient is a coefficient used to describe the degree of clustering between nodes in a network. Specifically, it is the degree of interconnection between adjacent points of a point. |
| local efficiency | The local efficiency of a node in the graph is the average global efficiency of the subgraph induced by the neighbors of the node. |
| betweenness centrality | Betweenness centrality is computed equivalently on weighted and directed networks, provided that path lengths are computed on respective weighted or directed paths. |
| eigenvector centrality | Eigenvector centrality is used to measure the level of influence of a node within a network. Each node within the network will be given a score or value: the higher the score the greater the level of influence within the network. This score is relative to the number of connections a node will have to other nodes. |
| pagerank centrality | The pagerank is a variant of the Eigenvector centrality score, but because it uses backlinks/in-degrees it is used in directed networks. |
| eccentricity | Eccentricity of node is used to represent the maximum distance between node in the graph and other nodes in the graph. |

**2.Details of Dimension reduction**

First, the minimum redundancy maximum relevance (mRMR) algorithm was used to extract robust features from the dataset. The aim of the the minimum redundancy process ensured that the selected features had minimal redundancy among the other features. At the same time, maximum relevance procedure was to select features having the maximum correlation with the actual grading of Progress in mild cognitive impairment，and we selected features with correlation coefficients greater than 0.8 and 0.1 as high correlation and low redundancy features, respectively. Then, the mRMR method was used to obtain an optimal feature set with a high correlation and low redundancy. Number of remained features: 544

Secondly, Dimensionality reduction of the selected 544 features was performed using the least absolute shrinkage and selection operator (LASSO) method. LASSO is a powerful algorithm for regression analysis with high dimensional predictors. The LASSO algorithm shrinks some coefficients and reduces others to exactly 0 via an absolute constraint. Thus, LASSO is an outstanding method for feature selection as it retains good features using both a subset selection and ridge regression. In this study, LASSO selected 28 nonzero coefficients. Finally, the gradient boosting decision tree (GBDT) algorithm was used to reduce the dimension of the remaining features.

GBDT is an algorithm that classifies or regresses data by the linear combination of basis functions and reduces the residual generated in the training process. In this study, five features were obtained from the GBDT procedur.

**3. Machine Learning Details**

In our study, we used support vector machines classifiers. The classifiers were implemented using R package caret [1], which provides a nice interface to access many machine-learning algorithms in R. Furthermore, it also provides a user-friendly framework for training different machine-learning models. We used parameter configurations of machine learning that were previously defined by Fernandez-Delgado et al [2], and we have listed the classification methods along with their parameters and corresponding R packages.

**Support vector machine (SVM)**

SVM, with Gaussian kernel function was implemented using a caret interface and R package kernlab. Cost parameter C was varied with values {2^-2^ , 2^-1^ ,1, 2^1^ , 2^2^ } and the parameter kernel spread was varied with values in {10^-2^ , 10^-1^ , 1, 10^1^ ,10^2^}.

**References**

1. Kuhn M. Building predictive models in R using the caret package. J Stat Softw. 2008, 28(5):1–26.
2. Fernández-Delgado, Cernadas, Barro, et al. Do we need hundreds of classifiers to solve real world classification problems? J. Mach. Learn. Res. 2014, 15:3133–3181.
